# Diabetes self-care practices and resilience in the Brazilian COVID-19 pandemic: results of a web survey: DIABETESvid

**DOI:** 10.1186/s13098-021-00706-8

**Published:** 2021-08-19

**Authors:** Bárbara Aparecida Binhardi, Carla Regina de Souza Teixeira, Bianca de Almeida-Pititto, Francisco Barbosa-Junior, Laercio Joel Franco, Karla Fabiana Santana de Melo, Domingos Augusto Cherino Malerbi, Rinaldo Eduardo Machado de Oliveira

**Affiliations:** 1 University of São Paulo, Ribeirão Preto College of Nursing, Av. Bandeirantes, 3900, Monte Alegre, Ribeirão Preto, São Paulo, CEP: 14040-902 Brazil; 2 Department of Preventive Medicine, Paulista School of Medicine, Universidade Federal de São Paulo, Rua Botucatu, No. 740, Vila Clementino, São Paulo, SP CEP: 04023-062 Brazil; 3 Brazilian Diabetes Society (SBD), Rua Afonso Braz, 579, Salas 72/74, Vila Nova Conceição, São Paulo, SP CEP: 04511-011 Brazil; 4 Ribeirão Preto Medical School, University of São Paulo, Av. Bandeirantes, 3900, Monte Alegre, Ribeirão Preto, São Paulo, CEP: 14049-900 Brazil; 5 Faculty of Medicine Clinics Hospital, University of São Paulo, Av. Dr. Enéas Carvalho de Aguiar, 255, Cerqueira César, São Paulo, 05403-000 Brazil; 6 Albert Einstein Israelite Hospital, Av. Albert Einstein, 627-Jardim Leonor, São Paulo, SP 05652-900 Brazil

**Keywords:** Diabetes mellitus, COVID-19, Self-care, Psychological resilience, Pandemic, Public health, Type 1 diabetes mellitus, Type 2 diabetes mellitus, Chronic disease, Comprehensive healthcare

## Abstract

**Background:**

The world guidance on the measures of social distancing for prevention of COVID-19 has changed the daily habits of great part of the population, and this could influence the care and resilience with diabetes during situations of adversity. This study aimed at assessing the characteristics of diabetic individuals and self-care practices and resilience with diabetes in the context of the COVID-19 pandemic in Brazil.

**Methods:**

This is a cross-sectional web survey study carried out among adults with diabetes, in which a structured 43-item questionnaire was conducted on the REDCap plataform, including the Diabetes Self-Care Activities Questionnaire and Connor-Davidson Resilience Scale, to measure socio-demographic and clinical characteristics. The web survey was disseminated through the main social media and data were collected from September 1st to October 19th, 2020. Data analysis was performed according to type of diabetes mellitus (DM) and at a significance level of 5% (p < 0.05).

**Results:**

Of the 1633 participants, 67.5% were women, 43.2% aged between 35 and 59 years old, 68.0% lived in the south-eastern region of Brazil, 57.1% had a high education level, 49% reported to have DM1 and 140 participants reported to have had COVID-19. Diabetes care mostly involved the use of medications (93%), whereas the least used ones were physical activity (24.6%) and examination of the shoes (35.7%). About 40% of the participants reported to be followed up by telemedicine, 61.5% monitored the glycaemic levels, 61.2% followed a healthy diet and 43.4% left home only to go to the supermarket and drugstore. The mean resilience was 25.4 (SD = 7.7).

**Conclusions:**

In Brazil, individuals with diabetes followed social distancing and maintained their medication treatment for DM. However, practice of physical activity and foot examination was little followed by the participants, who also had a low level of resilience. These findings showed the importance of patient follow-up in the healthcare services, meaning that telemedicine should be improved and support provided for adaptation in view of the therapeutic setbacks.

**Supplementary Information:**

The online version contains supplementary material available at 10.1186/s13098-021-00706-8.

## Background

The current pandemic of COVID-19 has become a great threat to the global health, involving 192 countries and regions and resulting in more than 2 million deaths worldwide up to the present day (February 2021) [1]. As well as Brazil, many countries entered social isolation or distancing by reducing travelling and activities to limit the spread of SARS-CoV-2 and allow the healthcare system to be better organized for more severe cases of COVID-19 [2–4].

People with chronic diseases such as diabetes mellitus (DM), obesity, hypertension or cardiovascular disease have a worse prognosis of COVID-19. According to a meta-analysis of 33 studies with 16,003 individuals, persons with diabetes are twice more likely to have their comorbid conditions worsened, and consequently to die, compared to those with no morbidity [5]. In addition, studies suggest that inadequate glycaemic control increases the risk of undesired outcomes in diabetes [6, 7].

Brazil has up to 16.8 million people with diabetes (11.4% of the population aged 20–79 years old), being among the five main countries in number of individuals with DM [8] as there is evidence that about 50% of them do not know they have the disease [9]. Additional data on glycaemic control in Brazil show that only a minority (about 25%) met the therapeutic goal of glycated hemoglobin (HbA1c) < 7% before the pandemic, as recommended by the Brazilian Diabetes Society (*SBD*) [10].

The world guidance on the measures of social distancing for prevention of COVID-19 is particularly aimed at vulnerable people, including those with DM. DM is a chronic condition demanding self-management and multidisciplinary care [11], which involve s regular attention to food, physical activity, blood sugar monitoring, use of medications and care with feet [12]. Moreover, resilience is considered the capacity to overcome adverse situations, in which the individual becomes strengthened or transformed by them [13]. Studies show a direct association between resilience and adequate care for DM [14, 15].

Social distancing during the COVID-19 pandemic can increase the risk of poor nutrition and reduce the physical activity, both related to mental health concerns, while the search for care is delayed due to the fear of contracting COVID-19 [16, 17]. On the other hand, this situation may have sensitized some people to the care with DM and thus accelerated the implementation of other types of healthcare, such as telemedicine [18]. Local, social, and demographic characteristics should be taken into consideration in strategies aimed to control the pandemic by managing the chronic diseases. In this context, there are some peculiarities in Brazil (e.g. heterogeneous population distribution across the country, cultural and geographic differences, striking social inequalities and difficult access to the healthcare services) which can influence the adherence to interventions for chronic diseases, such as DM [19].

In this context, it is crucial to understand the level of diabetes care in view of the measures adopted during the COVID-19 pandemic to elaborate control and preventive strategies. Therefore, this study was aimed at assessing the characteristics of diabetic people, including self-care practices with DM and resilience in the context of the COVID-19 pandemic in Brazil.

## Methods

This is a web survey conducted in the period between 1st September and 19th October 2020, in which the Checklist for Reporting Results of Internet E-Surveys (CHERRIERS) guidelines were used to ensure the quality and interpretation of the data [20, 21]. The sample was of convenience and the data were collected by means of an on-line questionnaire, conducted on the Research Electronic Data Capture (REDCap) platform hosted at https://redcap.fmrp.usp.br [22], in which 43 structured questions were completed anonymously. The web survey was disseminated on social networks (i.e. Facebook and Instagram) and made available to diabetes-related groups through link and QR-Code, including on-line conversation applications (i.e. WhatsApp and Telegram), websites of diabetes-related institutions, radio and television. It was estimated that the questionnaire completion time was, on average, 10 min.

The inclusion criteria were the following: accepting the conditions listed in the free informed consent form, reporting to have DM, living in Brazil and being older than 18 years old. If one of these three questions was negatively answered, the questionnaire was finished.

The questionnaire comprised socio-demographic items: gender, age, region of residence, education level; clinical data: time of DM diagnosis, type of DM (Type 1, Type 2 and “other types”—which included gestational diabetes, MODY, LADA, diabetes associated with pancreatic diseases and no information), medical diagnosis of COVID-19, testing for COVID-19, medications used and lack of any medication, site for delivery of medications (governmental pharmacies, “Farmácia Popular do Brasil” program, private pharmacies and drugstores and non-governmental organizations), self-reported diabetes control and self-reported alcoholic beverage consumption and therapeutic follow-up during the pandemic (i.e. type of healthcare service, healthcare insurance, telemedicine appointments, professional consulted, social isolation/distancing). Two other validated instruments were also used to measure self-care and resilience, namely, Diabetes Self-Care Activities Questionnaire (DSCA) [23] and Connor-Davidson Resilience Scale (CD-RISC-10) [24].

DSCA is a questionnaire translated into Portuguese and adapted to Brazil from the Summary of Diabetes Self-Care Activities Questionnaire. It has six domains and 15 items for assessment of the diabetes self-care as follows: general food, specific food, physical activity, glycaemic monitoring, use of medications and foot care. The three items on smoking habits were not included. DSCA is based on the number of days per week in which the respondent has a given behavior, with each item scoring from 0 to 7 points (i.e. 0 is the least desirable situation and 7 is the most desirable one). In the domain on specific food, the scores for items on consumption of fat-rich food and sweets are inverted. It was established that at least five days for each self-care activity is adequate [25, 26].

CD-RISC-10 is a self-administered instrument consisting of 10 items, with the answers scoring from 0 (never true) to 4 (always true). The result is the sum of the scores of each item, ranging from 0 to 40 points, in which higher scores suggest more resilience and lower scores suggest less resilience or more difficult in overcoming setbacks [27].

### Statistical analysis

The resulting data were stored on the REDCAp platform and exported in CSV format so that a descriptive analysis could be performed by using the Statistica for Windows software, version 13. The categorical variables were presented as numeric and percentage values. Chi-square test was used to compare the variables of interest depending on the type of diabetes as follows: type 1 diabetes mellitus (DM1), type 2 diabetes mellitus (DM2) and other types of diabetes. Statistical significance was set at p < 0.05 and confidence interval at 95%. Student t-test was used for comparative analysis between groups according to the types of diabetes.

## Results

A total of 3074 participants had access to the web survey, and 37.6% opted for not answering it. Of the 1918 participants who answered the questionnaire, 14.5% were excluded from the study due to the following reasons: 12.0% had no diabetes, 0.8% did not live in Brazil and 1.9% were younger than 18 years old. Therefore, 1633 diabetic people participated in this study: 49.3% had type 1 diabetes mellitus (DM1) and 39.5% type 2 diabetes mellitus (DM2). In addition, other types of diabetes were observed: 0.3% gestational diabetes, 0.7% MODY-type diabetes, 2.7% LADA-type diabetes, 0.5% diabetes associated with pancreatic disease, 1.0% “other types”, 5.4% did not know the type of diabetes they have, and 1.5% did not inform.

Table 1 shows the socio-demographic characteristics of the participants according to different types of DM. The final sample consisted of 1633 participants, with the majority being women (67.5%) and DM1 (49.3%). The majority of participants’ age ranged from 35 to 59 years, had higher education level (i.e. undergraduate and post-graduate degrees) and was living in the south-eastern region of the Brazil.

**Table 1:** Numeric and percentage distribution of the socio-demographic variables of the participants of the study

	Total		Type 1 diabetes		Type 2 diabetes		Other types		*P* value*
	n = 1633	%	n = 805	%	n = 628	%	n = 200	%	
Gender									
Female	1103	67.5	559	69.4	411	65.5	133	66.5	
Male	504	30.9	243	30.2	217	34.5	44	22.0	
Not informed	26	1.6	3	0.4	–	–	23	11.5	
Age group (years)									** < 0.0001**
18–34	575	35.2	503	62.5	33	5.25	39	19.5	
35–59	706	43.2	273	33.9	336	53.5	97	48.5	
≥ 60	352	21.6	29	3.6	259	41.2	64	32.0	
Region of the Country									**0.0013**
Northern	80	4.9	32	4.0	36	5.7	12	6.0	
North-eastern	184	11.3	83	10.3	86	13.7	15	7.5	
Centre-western	93	5.7	50	6.2	34	5.4	9	4.5	
South-eastern	1111	68.0	535	66.5	426	67.8	150	75.0	
Southern	165	10.1	105	13.0	46	7.32	14	7.0	
Education level									
Never attended school	4	0.2	–	–	4	0.6	–	–	
Elementary, incomplete	60	3.7	7	0.9	43	6.9	10	5.0	
Elementary, complete	65	4.0	8	1.0	44	7.0	13	6.5	
High school, incomplete	36	2.2	11	1.4	19	3.0	6	3.0	
High school, complete	215	13.1	98	12.2	93	14.8	24	12.0	
Technician courses	62	3.8	31	3.8	17	2.7	14	7.0	
College degreee, incomplete	231	14.2	171	21.2	42	6.7	18	9.0	
College degreee, complete	435	26.6	221	27.5	174	27.7	40	20.0	
Post-graduate education	498	30.5	256	31.8	191	30.4	51	25.5	
Not informed	27	1.7	2	0.2	1	0.2	24	12.0	

DIABETESvid, Brazil, 2020 (n = 1633)

*Pearson’s Chi-square test, comparing DM1 vs DM2 vs “other types”

Table 2 shows that the time of diabetes diagnosis of people with DM1 was very similar in the three ranges categories of 1–10 years (26.0%), 11–20 (35.8%) and ≥ 21 years (34.8%), while people with DM2 reported a higher frequency of diagnosis of 1–10 years (59.2%) than 11–20 years (23.4%) and ≥ 21 years (9.2%) and “other types” had similar proportions of DM2. Important to notice that for diagnosis less than 1 year, the higher frequency was of “other types” (17.5%), followed by DM2 (8.1%) and DM1 (3.3%).

**Table 2:** Numeric and percentage distribution of the clinical variables of the participants regarding diabetes and COVID-19 (n = 1633)

	Total		Type 1 diabetes		Type 2 diabetes		Other types		*P* value*
	n = 1633	%	n = 805	%	n = 628	%	n = 200	%	
Time of diabetes diagnosis (years)									
< 1	113	6.9	27	3.3	51	8.1	35	17.5	
1–10	667	40.9	209	26.0	372	59.2	86	43.0	
11–20	472	28.9	288	35.8	147	23.4	37	18.5	
≥ 21	356	21.8	280	34.8	58	9.2	18	9.0	
Not informed	25	1.5	1	0.1	–	–	24	12.0	
Medical/clinical diagnosis of COVID-19	135	8.3	64	7.9	58	9.2	13	6.5	** < 0.0001**
Testing for COVID-19	437	26.8	219	27.2	172	27.4	46	23.0	** < 0.0001**
Test result for COVID-19 (n = 437)									** < 0.0001**
Positive	120	25.8	57	25.8	52	29.7	11	15.7	
Negative	310	66.5	157	71.0	119	68.0	34	48.6	
No result received	7	1.5	5	2.3	1	0.6	1	1,4	
Hospitalization due to COVID-19 (n = 140)	42	30.0	19	28.8	23	37.7	–	–	

*Pearson’s chi-square test, comparing DM1 vs DM2 vs “other types”

About COVID-19, of the 135 clinical diagnosed cases, 115 had laboratory confirmation for SARS-CoV-2. In addition to these cases, five cases had laboratory confirmation despite the lack of medical diagnosis. DM2 had higher frequency of clinical diagnosis (9.2%) compared to DM1 (7.9%) and “other types” (6.5%). Regarding positive tests for Covid-19, it was seen in 29.7% of DM2, 25.8% of DM1 and 15.7% of “other types” participants. In total, there were 140 cases of COVID-19 (8.6% of the total sample) and of these 30.0% needed hospitalization, with greater frequency of DM2 with COVID-19 reporting hospitalization (37.7%) than DM1 (28.8%).

Of the 113 participants reporting to be diagnosed with DM within 1 year, 17 had COVID-19 and of these 11 were diagnosed with DM after contracting COVID-19, with 9 being hospitalized.

Considering the therapeutic follow-up during the COVID-19 pandemic, Table 3 shows that 68.7% had health care insurance and that 52.4% were followed up in private health care services, 18.0% in public primary care settings and 6.9% in public specialized clinics. From the 856 who reported assistance in private care system, 79 also referred assistance in public health care. Considering the public health system, we observed that DM1 referred to be followed-up more in specialized clinics, while DM2 was more in primary care assistance. From all individuals, 27.9% reported having no follow-up assistance since March 2020. From those with DM1 this percentage was 22.5%, from DM2 it was 34.5% and from 'other types' it was 28.5%.

**Table 3:** Numeric and percentage distribution of the participants regarding their therapeutic follow-up during COVID-19 pandemic (n = 1633)

	Total		Type 1 diabetes		Type 2 diabetes		Other types		*P* value*
	n = 1633	%	n = 805	%	n = 628	%	n = 200	%	
Have Private healthcare insurance	1121	68.65	596	74.0	412	65.6	113	56.5	** < 0.0001**
Types of follow-up healthcare services for diabetes									
Public Primary Care	294	18.0	136	16.9	121	19.3	37	18.5	**0.5006**
Public specialised outpatient clinics	113	6.9	82	10.2	22	3.5	9	4.5	** < 0.0001**
Private healthcare service or private healthcare insurance	856	52.4	480	59.6	288	45.9	88	44.0	** < 0.0001**
No presencial appointments since March 2020	455	27.9	181	22.5	217	34.5	57	28.5	** < 0.0001**
Had telemedicine appointments	518	31.7	319	39.6	160	25.5	39	19.5	** < 0.0001**
Professionals offering telemedicine services (n = 518)									
Physician	476	29.1	294	36.5	144	22.9	38	19.0	** < 0.0001**
Nurse	37	2.3	26	3.2	8	1.3	3	1.5	**0.0351**
Pharmacyst	45	2.8	22	2.7	21	3.3	2	1.0	**0.2109**
Nutritionist	89	5.4	61	7.6	17	2.7	11	5.5	**0.0003**
Physical educator	21	1.3	15	1.9	3	0.5	3	1.5	**0.0666**
Psychologist	49	3.0	38	4.7	10	1.6	1	0.5	**0.0002**
Physiotherapist	6	0.4	3	0.4	2	0.3	1	0.5	**0.9334**
Other health professionals	23	1.4	11	1.4	10	1.6	2	1.0	**0.8173**
Medications used for diabetes control									
Insulin(s)	999	61.2	778	96.6	134	21.3	87	43.5	** < 0.0001**
Tablet(s)	779	47.7	87	10.8	586	93.3	106	53.0	** < 0.0001**
Non-insulin injetable medication	45	2.8	10	1.2	32	5.1	3	1.5	** < 0.0001**
No medication for diabetes	22	1.3	2	0.2	10	1.6	10	5.0	** < 0.0001**
Site for delivery of diabetes medications									
Governamental pharmacies	703	43.0	466	57.9	186	29.6	51	25.5	** < 0.0001**
“Farmácia Popular do Brasil” Program	435	26.6	151	18.8	234	37.3	50	25.0	** < 0.0001**
Private pharmacies and drugstores	662	40.5	263	32.7	308	49.0	91	45.5	** < 0.0001**
Non-governmental organizations**	128	7.8	107	13.3	16	2.5	5	2.5	** < 0.0001**
Do not know	5	0.3	2	0.2	2	0.3	1	0.5	**0.8449**
Lack of any medication for diabetes during pandemic	161	9.9	102	12.7	45	7.2	14	7.0	** < 0.0001**

*Pearson’s chi-square test, comparing DM1 vs DM2 vs “other types”

**Churches, associations and labour unions

In the pandemic 31.7% reported telemedicine appointments, which were more frequent in DM1 than in DM2 participants. Physicians were the professionals more frequently consulted, followed by the nutritionists and psychologists in the total sample and according to the type of diabetes.

As expected, insulin was the medication most used by the participants with DM1 and oral medications by those with DM2. In the “other types” of diabetes group we find almost the same frequency of insulin and oral medication use and observed 5% without any medications for diabetes.

The public healthcare system were the main sites for delivery of medications during the pandemic. Some patients got their medication from both sites of public health system: the primary health care units and popular pharmacy program. Together, the primary health care units and the popular pharmacy program accounted for 62.2% of the sites for delivery of medications in total sample, being slightly higher in DM1 (76.7%) than in DM2 (66.9%) and “other types” of diabetes (50.5%) groups.

Lack of medication was reported by 161 (9.9%) of the total participants, with a higher frequency in DM1 (12.7%) than in DM2 (7.2%) or “other types” (7.0%) groups.

From those who referred lack of any diabetes medication during the pandemic period of the study, we observed that 61.1% reported public health system as the site where they got their medication from, followed by 19.7% from private drugstores, 12.7% from both sites (public and private) and 6.4% from “other sites” (p = 0.032).

Table 4 shows that 44.6% of the participants had their daily routine changed, such as leaving home for essential activities and work only. The proportion of social distance categories were similar according to the type of diabetes.

**Table 4:** Numeric and percentage distribution of the participants regarding changes in daily routine during COVID-19 pandemic (n = 1633)

	Total		Type 1 diabetes		Type 2 diabetes		Other types		*P* value*
	n = 1633	%	n = 805	%	n = 628	%	n = 200	%	
Social distancing									** < 0.0001**
Leaving home for healthcare only	362	22.2	181	22.6	143	23.0	38	21.5	
Leaving home to go to supermarket and drugstore only	728	44.6	356	44.5	290	46.6	82	46.3	
Leaving home to work, with little decrease in social contact	457	28,0	244	30.5	162	26.0	51	28.8	
Done nothing at all, leading a normal life	52	3.2	19	2.4	27	4.3	6	3.4	
Did no answer	34	2.1	5	0.6	6	1.0	23	11.0	
Changes in diabetes control during the pandemic									** < 0.0001**
Diabetes control remained the same	707	43.0	278	34.5	348	55.4	81	40.5	
Diabetes control worsened a bit	435	26.6	244	30.3	141	22.5	50	25.0	
Diabetes control worsened a lot	184	11.3	98	12.2	65	10.3	21	10.5	
Diabetes control improved a bit	185	11.3	126	15.6	47	7.5	12	6.0	
Diabetes control improved a lot	87	5.3	57	7.1	20	3.2	10	5.0	
Did not answer	35	2.1	2	0.3	7	1.1	26	13.0	
Consumption of alcoholic beverage after onset of the pandemic									** < 0.0001**
Do not consume alcoholic beverage	884	55.1	391	48.6	385	61.3	108	61.4	
Consumed alcoholic beverage before, but not during the pandemic	82	5.1	53	6.6	20	3.2	9	5.1	
Continued consuming alcoholic beverage at the same frequency	314	19.6	161	20.0	115	18.3	38	19.6	
Alcoholic beverage consumption increased	123	7.7	71	8.8	42	6.7	10	5.7	
Alcoholic beverage consumption decreased	200	12.5	124	15.4	65	10.3	11	6.3	
Did not answer	30	1.8	5	0.6	1	0.2	24	12.0	

*Pearson’s chi-square test, comparing DM1 vs DM2 vs “other types”

Regarding diabetes control, 37.9% of the participants reported that the control of the disease worsened. According to type of diabetes, 55.4% of DM2 referred that their control remained the same, proportion higher than for DM1 (43%) or “other types” (40.5%). In fact, a greater percentage of DM1 participants reported that their glucose control was worse than previous to the pandemic period when compared to DM2 and “other types” group: 42.5% for DM1, 32.8% for DM2 and 35.5% for “other types”. The proportion of participants reporting that the control of diabetes improved during this period was 22.7% for DM1, 10.7% for DM2 and 11.0% for “other types”.

It was observed that 115 (18.3%) participants reported consumption of alcoholic beverage with the same frequency, whereas 42 (6.7%) reported increase during the pandemic (DM1 8.8%, DM2 6.7% and “other types” 5.0%).

**Fig. 1:**
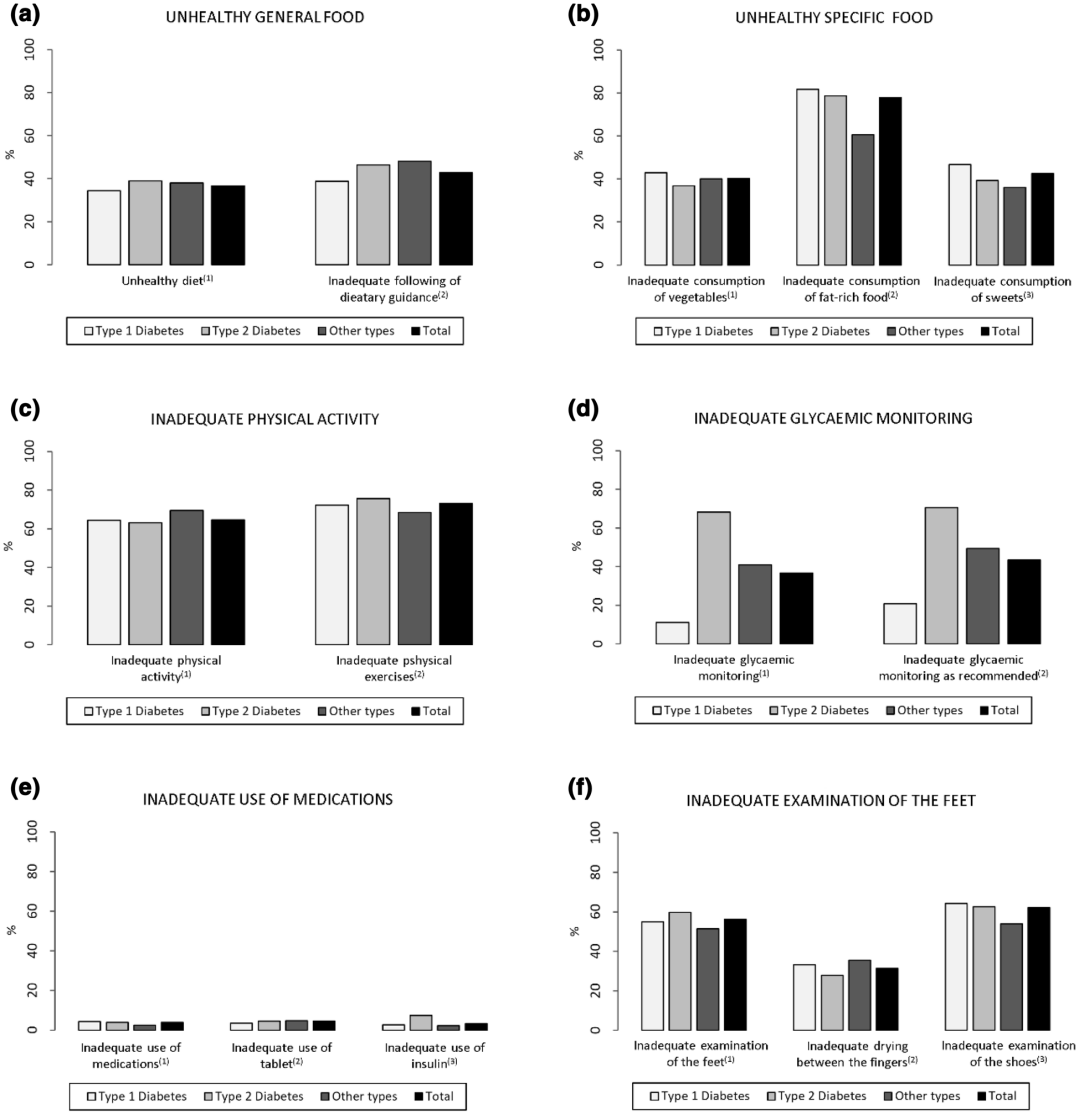
Percentage distribution of the participants regarding inadequate diabetes self-care during COVID-19 pandemic (n = 1633). Percentage of participants in relation to inappropriately performed diabetes self-care activities, investigated by the questions: **a1**. How many of the last seven days have you followed a healthful eating plan?/**a2**. On average, over the past month, how many days per week have you followed your eating plan?/**b1**. On how many of the last seven days did you eat five or more servings of fruits and vegetables?/**b2**. On how many of the last seven days did you eat high fat foods such as red meat or full-fat dairy products?/**b3**. On how many of the last seven days did you eat sweets?) **c1** On how many of the last seven days did you participate in at least 30 min of physical activity?/**c2**. On how many of the last seven days did you participate in a specific exercise session?/**d1**. On how many of the last seven days did you test your blood sugar?/**d2**. On how many of the last seven days did you test your blood sugar the number of times recommended by your health care provider?/**e1**. On how many of the last seven days, did you take your recommended diabetes medication?/**e2**. On how many of the last seven days did you take your recommended number of diabetes pills?/**e3**. On how many of the last seven days did you take your recommended insulin injections?/**f1**. On how many of the last seven days did you examinate your feet?/**f2**. On how many of the last seven days did you dry between your toes after washing?/**f3**. On how many of the last seven days did you examine inside your shoes? (For more information, see “Additional file 1” at the end of this file)

Regarding diabetes self-care, Fig. 1 shows that most of the participants reported maintaining a healthy diet (61.2%), following dietary guidance (54.9%), with an adequate frequency of vegetables consumption (57.9%), but with high frequencies of fat-rich food (77.9%) and sweets (42.6%) consumption.

According to type of diabetes, those with DM1 reported more frequently an adequate healthy diet, following dietary guidance. When the questionnaire addressed specific foods, DM2 had higher frequencies of adequate consumption of vegetables and fruits, fat-rich foods, and sweets.

Inadequate frequencies of physical activity (64.6%) and physical exercise (73.1%) were reported by participants, with similar results according to type of diabetes. Physical activity was more frequent in DM1 and DM2 than “other types” of diabetes, while physical exercise in leisure time was not different among the three groups and was very low in all of them.

Monitorization of glycaemic levels according to professionals’s recommendations was reported by 79.0% of the DM1, 27.7% of the DM2 and 39.0% of “other types” groups (p < 0.001).

Use of medication adequately was reported by 95% of DM1, 94.3% of DM2 and 80.5% of “other types” groups.

We found that 920 (56.3%) of the participants reported not performing foot examination.

With regard to resilience, CD-RISC-10 was used among 1543 participants, and we found a mean score of 25.4 (SD = 7.7). In the group of participants with DM1, the mean score was 24.7 (SD = 7.6) and in the group of those with DM2, the mean score was 26.4 (SD = 7.6). Participants with other types of diabetes had a mean score of 25.0 (SD = 7.7).

## Discussion

In this web survey we addressed characteristics of people with diabetes that attended the questionnaire and how they reported self-care and resilience during a period of the COVID-19 pandemic, calling attention to low levels of behavioral components of this self-care and worrying scores in the resilience questionnaire.

The sample of this study consisted of 1633 diabetic individuals from all regions of Brazil, with the majority living in the south-eastern of the country. Of the 628 participants with DM2, 53.5% were aged between 35 and 59 years and from 805 with DM1, 62.5% were aged between 18 and 34 years. A Brazilian survey tool shows that 74% of the Brazilian people use Internet, but this percentage drops to 57% in lower socio-economic classes (i.e. D and E) (*TIC Domicílios*, 2019). In terms of region, south-eastern is the one with the highest percentage of homes with access to Internet [28]. In January 2021, according to the Statistica software, more than 31% of the Instagram users in Brazil are aged between 25 and 34 years, with women comprising the majority of users of this social network application [29].

About type of diabetes, DM1 was the predominant type of diabetes reported by the participants (49.3%), following by DM2 (39.5%) and a variety of other types of diabetes, including the response that they did not know de type they presented (5.4%), and probably some misclassifications may occur since there are 27 reports of being DM1 but not in use of insulin. Our findings regarding types 1 and 2 of diabetes were similar from those reported by a study on the challenges faced by Brazilians during the pandemic (i.e. from 22 April to 4 May 2020), with the majority from type 1 (60.73% had DM1), but with less type 2 than our study (30.75% had DM2) [19]. Interesting to notice that 5.4% of the participants did not know the type of diabetes they have, and this situation is distributed in all the levels of education categories.

In this sample, 6.9% of the participants reported a diagnosis of DM of less than one year. Interestingly, 11 participants reported being diagnosed with DM during infection by SARS-CoV-2 or immediately after contracting the virus. It is already known that COVID-19 would act as a trigger for the diagnosis of diabetes, DM1, DM2 or any other type of diabetes mentioned in this study, by specific mechanisms affecting glycemia and sub-clinical inflammation. On the other hand, there is a hypothesis that the virus may have a diabetogenic potential, which is not clear yet and is currently being investigated by some studies [30].

It is relevant to observe that most of the participants (44.6%) followed restrictions for COVID-19 by leaving home only to go to supermarkets and drugstores and this finding is in accordance with other studies and national researches. The National Household Sample Survey (*PNAD* COVID-19) conducted by the Brazilian Institute of Geography and Statistics (*IBGE*) found that the percentage of people reporting leaving home for basic needs was 40.5% of the population (i.e. 85.6 million people) [31]. Another Brazilian study on COVID-19 [19] about behaviour of people with diabetes in the beginning of the pandemic, showed that there were changes in their daily habits, mainly regarding to a decrease in the frequency of leaving home, a finding also observed in our study.

Considering the follow-up of diabetes, 52.4% participants reported assistance in private healthcare services and 24.9% in the public healthcare system, which is in accordance with the sociodemographic profile of our sample with a high frequency of high education level that is a proxy for socioeconomic status. We call attention for the finding that 27.9% had no presential follow-up between March and October 2020; from which, DM2 represented 47.7% and DM1 39.8% of the individuals without appointments during this period of the pandemic. The Pan-American Health Organization (PAHO/WHO) recommends that countries ensure that people with diabetes receive full treatment during the pandemic. This may involve providing care out of the traditional settings by using virtual health resources to disseminate information and maximize health care services for the population by means of community health agents [32]. Studies show that the follow-up of people with diabetes by using telemedicine is favourable as it enables the detection of clinical and psychological needs and provides support to the patients, including significant improvement in glycaemic levels [33, 34]. In our sample, 31.7% participants receive care through telemedicine, and of these, 39.6% were individuals with DM1. Of those who reported no presential appointments during March to October of 2020, only 12.4% had some telemedicine orientation (data not shown).

In our findings, although most of the participants reported following a healthy diet (61.2%) and consuming adequate food (54.9%), the consumption of fat-rich foods (77.9%) and sweets (42.6%) was inadequate. Also, we observed a relevant percentage of inadequate physical activity (64.6%). A recent study showed an increase in physical inactivity in about 28% of the adults during the COVID-19 pandemic [35]. The damages to diabetic individuals resulting from the reduced physical activity during the pandemic, such as poor metabolic and hypertension control, were reported by a review study in which the authors also estimated increases in the physical inactivity in cases of DM2 (from 7.2% to 9.6%) and in the mortality resulting from all causes (from 9.4% to 12.5%) across the world [36]. The decrease in physical activity, in association with an increase in the consumption of some fat- and carbohydrate-rich food, is being related to body weight gain and poor metabolic control [37–39].

Diabetes self-care adherence was greater for use of medications and consumption of healthy diet, but it was lower for glycaemic monitoring, physical activity and foot examination, which were frequently reported findings even before the pandemic [40]. The Diabetes Attitudes, Wishes and Needs second study (DAWN2), an international survey conducted in 2015 to assess the general and self-care management for diabetes among 8596 diabetic people from 17 countries, showed that advice on self-care was more common for medications and diet, whereas those for glycaemic monitoring, physical activity and foot examination were the less common [41].

In the context of the pandemics, individuals with chronic conditions might end up facing challenges due to the poor diabetes self-care management, among other daily demands [42]. Therefore, resilience emerges as a mechanism of adaptation to the COVID-19 pandemic [43].

As cited earlier, the mean score obtained in this study by means of the CD-RISC-10 was 25.4 (SD = 7.7). By comparing this figure to those of pre-pandemic studies, one can observe that this mean score is lower than those reported by a Brazilian study with a general population (29.1; SD = 5.5) and by a Chinese study with diabetic individuals (30.7; SD = 5.8) [44]. Our mean score is very close to that found by a previous Brazilian study with psychiatric outpatients followed up before the COVID-19 pandemic, namely, 25.8 (SD = 9.1) [44].

During the peak of COVID-19 pandemic in China between February and March 2020, it was found that resilience in general population was negatively associated with symptoms of depression and anxiety, which are important factors in the control of diabetes [42, 45].

Resilience was one of the topics addressed during the International COVID-19 and Diabetes Virtual Summit sponsored by the Diabetes Technology Society in collaboration with Sansum Diabetes Research Institute aiming to protect diabetic people from the severe effects of COVID-19. Thus, resilience is thought to be a gap of knowledge to be explored as even after vaccination, diabetic individuals can still face many difficulties [46].

Some limitations of this study were due to the inequality in the access to Internet in Brazil and the self-reported diagnosis of diabetes. The type of diabetes was self-reported and could occur some misleading classification of type of diabetes. Diabetes control data was self-reported and not based in glycaemic or HbA1c values.

## Conclusions

In Brazil, diabetic people during the COVID-19 pandemic reported they followed the social distancing rules and maintained the care with diabetes, but they reduced the practice of physical activity and the examination of the feet, in addition to the low level of resilience. Among the diabetic groups, there was a statistically significant difference between all variables regarding self-care and resilience. These findings show the importance of following up patients in the healthcare services, meaning that telemedicine should be improved, and support provided for adaptation in view of the therapeutic setbacks. New studies are necessary to monitor the behaviour of diabetes in the long-term regarding self-care and resilience with the progression of COVID-19 pandemics.

## Supplementary Information

**Additional file 1: **Numeric and percentage distribution of the participants regarding diabetes self-care during COVID-19 pandemic (n = 1633)”. The table shows the frequency of self-care behaviors of Brazilians with diabetes during the COVID-19 pandemic, using the DSCA instrument. DSCA is a questionnaire translated into Portuguese and adapted to Brazil from the Summary of Diabetes Self-Care Activities Questionnaire. It has six domains and 15 items for assessment of the diabetes self-care as follows: general food, specific food, physical activity, glycaemic monitoring, use of medications and foot care. DSCA is based on the number of days per week in which the respondent has a given behavior, with each item scoring from 0 to 7 points (i.e. 0 is the least desirable situation and 7 is the most desirable one). In the domain on specific food, the scores for items on consumption of fat-rich food and sweets are inverted. It was established that at least five days for each self-care activity is adequate.

## Data Availability

The datasets used and/or analysed during the current study are available from the corresponding author on reasonable request.

## Abbreviations

ACE2: Angiotensin-converting enzyme-2
ADA: American Diabetes Association
CD-RISC-10: Connor-Davidson Resilience Scale
CHERRIES: Checklist for Reporting Results of Internet E-Surveys
COVID-19: 2019 Novel coronavirus
DM: Diabetes mellitus
DM1: Type 1 diabetes mellitus
DM2: Type 2 diabetes mellitus
DSCA: Diabetes Self-Care Activities Questionnaire
HbA1c: Glycated hemoglobin
IBGE: Brazilian Institute of Geography and Statistics
PAHO: Pan-American Health Organization
PNAD: National Household Sample Survey
REDCap: Research electronic data capture
SBD: Brazilian Society of Diabetes
SDSCA: Summary of Diabetes Self-Care Activities Questionnaire
SUS: Sistema Único de Saúde (Public Health System)
*TIC Domicílios*: Information and Communication Technologies—residences
WHO: World Health Organization
