# Supplementary material for: Diabetes self-care practices and resilience in the Brazilian COVID-19 pandemic: results of a web survey: DIABETESvid
Source: Diabetol Metab Syndr. 2021 Aug 19;13:87. doi: 10.1186/s13098-021-00706-8 (PMC8374417; doi:10.1186/s13098-021-00706-8)
Supplement: Supplementary file 1 — Additional file 1: Numeric and percentage distribution of the participants regarding diabetes self-care during COVID-19 pandemic (n = 1633)”. The table shows the frequency of self-care behaviors of Brazilians with diabetes during the COVID-19 pandemic, using the DSCA instrument. DSCA is a questionnaire translated into Portuguese and adapted to Brazil from the Summary of Diabetes Self-Care Activities Questionnaire. It has six domains and 15 items for assessment of the diabetes self-care as follows: general food, specific food, physical activity, glycaemic monitoring, use of medications and foot care. DSCA is based on the number of days per week in which the respondent has a given behavior, with each item scoring from 0 to 7 points (i.e. 0 is the least desirable situation and 7 is the most desirable one). In the domain on specific food, the scores for items on consumption of fat-rich food and sweets are inverted. It was established that at least five days for each self-care activity is adequate. [file 13098_2021_706_MOESM1_ESM.docx]

**Additional file 1**. Numeric and percentage distribution of the participants regarding diabetes self-care during COVID-19 pandemic (n = 1,633).

|  | **Total** | | **Type 1 Diabetes** | | **Type 2 Diabetes** | | **Other types** | | ***P* value*** |
| --- | --- | --- | --- | --- | --- | --- | --- | --- | --- |
|  | N=1,633 | % | N=805 | % | N=628 | % | N=200 | % |  |
| **GENERAL FOOD** |  |  |  |  |  |  |  |  |  |
| **Healthy diet, n=1596** |  |  |  |  |  |  |  |  | **< 0.0001** |
| Adequate | 999 | 61.2 | 523 | 65.0 | 380 | 60.5 | 96 | 48.0 |  |
| Inadequate | 597 | 36.6 | 277 | 34.4 | 244 | 38.9 | 76 | 38.0 |  |
| Did not answer | 37 | 2.3 | 5 | 0.6 | 4 | 0.6 | 28 | 14.0 |  |
| **Dietary guidance, n=1594** |  |  |  |  |  |  |  |  | **< 0.0001** |
| Adequate | 896 | 54.9 | 490 | 60.9 | 328 | 52.2 | 78 | 39.0 |  |
| Inadequate | 698 | 42.7 | 311 | 38.7 | 291 | 46.3 | 96 | 48.0 |  |
| Did not answer | 39 | 2.4 | 4 | 0.5 | 9 | 1.4 | 26 | 13.0 |  |
|  |  |  |  |  |  |  |  |  |  |
| **SPECIFIC FOOD** |  |  |  |  |  |  |  |  |  |
| **Consumption of vegetables, n=1603** |  |  |  |  |  |  |  |  | **< 0.0001** |
| Adequate | 946 | 57.9 | 456 | 56.6 | 394 | 62.7 | 96 | 48.0 |  |
| Inadequate | 657 | 40.2 | 346 | 43.0 | 231 | 36.8 | 80 | 40.0 |  |
| Did not answer | 30 | 1.8 | 3 | 0.4 | 3 | 0.5 | 24 | 12.0 |  |
| **Consumption of fat-rich food, n=1599** |  |  |  |  |  |  |  |  | **< 0.0001** |
| Adequate | 327 | 20.0 | 142 | 17.6 | 131 | 20.9 | 54 | 27.0 |  |
| Inadequate | 1272 | 77.9 | 657 | 81.6 | 494 | 78.7 | 121 | 60.5 |  |
| Did not answer | 34 | 2.7 | 6 | 0.8 | 3 | 0.5 | 25 | 12.5 |  |
| **Consumption of sweets, n=1601** |  |  |  |  |  |  |  |  | **< 0.0001** |
| Adequate | 905 | 55.4 | 426 | 52.9 | 376 | 59.9 | 103 | 51.5 |  |
| Inadequate | 696 | 42.6 | 377 | 46.8 | 247 | 39.3 | 72 | 36.0 |  |
| Did not answer | 32 | 2.0 | 2 | 0.3 | 5 | 0.8 | 25 | 12.5 |  |
|  |  |  |  |  |  |  |  |  |  |
| **PHYSICAL ACTIVITY** |  |  |  |  |  |  |  |  |  |
| **Physical activity, n=1602** |  |  |  |  |  |  |  |  | **< 0.0001** |
| Adequate | 547 | 33.5 | 283 | 35.2 | 226 | 36.0 | 38 | 19.0 |  |
| Inadequate | 1055 | 64.6 | 519 | 64.5 | 397 | 63.2 | 139 | 69.5 |  |
| Did not answer | 31 | 1.9 | 3 | 0.4 | 5 | 0.8 | 23 | 11.5 |  |
| **Physical exercises, n=1595** |  |  |  |  |  |  |  |  | **< 0.0001** |
| Adequate | 401 | 24.5 | 216 | 26.8 | 148 | 23.6 | 37 | 18.5 |  |
| Inadequate | 1194 | 73.1 | 582 | 72.3 | 475 | 75.6 | 137 | 68.5 |  |
| Did not answer | 38 | 2.3 | 7 | 0.9 | 5 | 0.8 | 26 | 13.0 |  |
|  |  |  |  |  |  |  |  |  |  |

* Pearson’s chi-square test, comparing DM1 vs DM2 vs “other types”.

**Additional file 1 (cont.)**. Numeric and percentage distribution of the participants regarding diabetes self-care during COVID-19 pandemic (n = 1,633).

|  | **Total** | | **Type 1 Diabetes** | | **Type 2 Diabetes** | | **Other types** | | ***P* value*** |
| --- | --- | --- | --- | --- | --- | --- | --- | --- | --- |
|  | N=1,633 | % | N=805 | % | N=628 | % | N=200 | % |  |
| **GLYCAEMIC MONITORING** |  |  |  |  |  |  |  |  |  |
| **Monitoring of the blood sugar levels, n=1604** |  |  |  |  |  |  |  |  | **< 0.0001** |
| Adequate | 1004 | 61.5 | 715 | 88.8 | 196 | 31.2 | 93 | 46.5 |  |
| Inadequate | 600 | 36.7 | 89 | 11.1 | 429 | 68.3 | 82 | 41.0 |  |
| Did not answer | 29 | 1.8 | 1 | 0.12 | 3 | 0.48 | 25 | 12.5 |  |
| **Monitoring of the blood sugar levels as recommended, n=1597** |  |  |  |  |  |  |  |  | **< 0.0001** |
| Adequate | 888 | 54.4 | 636 | 79.0 | 174 | 27.7 | 78 | 39.0 |  |
| Inadequate | 709 | 43.4 | 167 | 20.8 | 443 | 70.6 | 99 | 49.5 |  |
| Did not answer | 36 | 2.2 | 2 | 0.3 | 11 | 1.8 | 23 | 11.5 |  |
|  |  |  |  |  |  |  |  |  |  |
| **USE OF MEDICATION** |  |  |  |  |  |  |  |  |  |
| **Use of medications, n=1583** |  |  |  |  |  |  |  |  |  |
| Adequate | 1518 | 93.0 | 765 | 95.0 | 592 | 94.3 | 161 | 80.5 | **< 0.0001** |
| Inadequate | 65 | 4.0 | 35 | 4.4 | 25 | 4.0 | 5 | 2.5 |  |
| Did not answer | 50 | 3,1 | 5 | 0.6 | 11 | 1.8 | 34 | 17.0 |  |
| **Use of tablets, n=777** |  |  |  |  |  |  |  |  | **0.0113** |
| Adequate | 742 | 95.3 | 84 | 96.6 | 559 | 95.4 | 99 | 93.4 |  |
| Inadequate | 35 | 4.5 | 3 | 3.5 | 27 | 4.6 | 5 | 4.7 |  |
| Did not answer | 2 | 0.3 | - | - | - | - | 2 | 1.9 |  |
| **Use of insulin, n=996** |  |  |  |  |  |  |  |  | **0.0157** |
| Adequate | 963 | 96.4 | 756 | 97.2 | 123 | 91.8 | 84 | 96.6 |  |
| Inadequate | 33 | 3.3 | 21 | 2.7 | 10 | 7.5 | 2 | 2.3 |  |
| Did not answer | 3 | 0.3 | 1 | 0.1 | 1 | 0.8 | 1 | 1.2 |  |
|  |  |  |  |  |  |  |  |  |  |

* Pearson’s chi-square test, comparing DM1 vs DM2 vs “other types”.

**Additional file 1 (cont.)**. Numeric and percentage distribution of the participants regarding diabetes self-care during COVID-19 pandemic (n = 1,633).

|  | **Total** | | **Type 1 Diabetes** | | **Type 2 Diabetes** | | **Other types** | | ***P* value*** |
| --- | --- | --- | --- | --- | --- | --- | --- | --- | --- |
|  | N=1,633 | % | N=805 | % | N=628 | % | N=200 | % |  |
| **FOOT CARE** |  |  |  |  |  |  |  |  |  |
| **Examination of the feet, n=1600** |  |  |  |  |  |  |  |  | **< 0.0001** |
| Adequate | 680 | 41.6 | 358 | 44.5 | 249 | 39.7 | 73 | 36.5 |  |
| Inadequate | 920 | 56.3 | 442 | 54.9 | 375 | 59.7 | 103 | 51.5 |  |
| Did not answer | 33 | 2.0 | 5 | 0.6 | 4 | 0.64 | 24 | 12 |  |
| **Drying between the fingers, n=1604** |  |  |  |  |  |  |  |  | **< 0.0001** |
| Adequate | 1091 | 66.8 | 535 | 66.5 | 451 | 71.8 | 105 | 52.5 |  |
| Inadequate | 513 | 31.4 | 267 | 33.2 | 175 | 27.9 | 71 | 35.5 |  |
| Did not answer | 29 | 1.8 | 3 | 0.4 | 2 | 0.3 | 24 | 12.0 |  |
| **Examination of the shoes, n=1601** |  |  |  |  |  |  |  |  | **< 0.0001** |
| Adequate | 583 | 35.7 | 285 | 35.4 | 232 | 36.9 | 66 | 33.0 |  |
| Inadequate | 1018 | 62.3 | 517 | 64.2 | 393 | 62.6 | 108 | 54.0 |  |
| Did not answer | 32 | 2.0 | 3 | 0.4 | 3 | 0.5 | 26 | 13.0 |  |

* Pearson’s chi-square test, comparing DM1 vs DM2 vs “other types”.
